# A Rare Presentation of Juvenile Rectal Polyps in an Adolescent Male With Rectal Bleeding: A Case Report

**DOI:** 10.7759/cureus.84866

**Published:** 2025-05-27

**Authors:** Parlapothula Hemantha Kumar, Ashok Kumar, Anoop Sachi, Hetal G Koriya, Vikrant Sharma, Kandarp Saxena

**Affiliations:** 1 Department of Shalya Tantra, National Institute of Ayurveda, Jaipur, IND; 2 Department of Gastroenterology, National Institute of Ayurveda, Jaipur, IND

**Keywords:** colonic polyps, colonoscopy, haematochezia, juvenile polyposis syndrome, juvenile rectal polyps

## Abstract

Juvenile rectal polyps are hamartomatous growths commonly occurring in the colon and rectum, characterized by cystic dilation of glandular structures within the lamina propria. They are typically under 10 years of age, but may also be seen in adolescents. This case report describes a 13-year-old boy with a five-year history of difficulty in defecation, intermittent rectal bleeding that progressively worsened over the past year, along with abdominal pain and prolapse of a rectal mass. Initial evaluations by multiple clinicians misdiagnosed the condition as hemorrhoids, delaying the correct diagnosis. Colonoscopy revealed multiple large polyps in the lower rectoanal region. Surgical excision was performed, and histopathological examination confirmed the presence of juvenile rectal (retention) polyps with unusual features, including torsion and mucin extravasation. This case underscores the importance of considering juvenile polyps in the differential diagnosis of rectal bleeding in pediatric patients and highlights the necessity for early and accurate diagnosis to prevent complications. Histopathological analysis plays a crucial role in guiding the management and ensuring appropriate treatment.

## Introduction

Juvenile polyps are typically benign hamartomatous growths commonly found in the colon, particularly the rectosigmoid region [1]. They are more frequently observed in children aged between 3 and 10 years and may also occur in adolescents [2]. Juvenile polyps are more often solitary than multiple, usually pedunculated, and usually vary in size from 5 mm to 3 cm [2,3]. The prevalence in children ranges from 0.08% to 3.7%, with a higher incidence in boys [4].

Common clinical presentations include painless rectal bleeding, abdominal pain, altered bowel habits, and occasional protrusion of a mass per anum [3]. Although the exact etiology remains unclear, juvenile polyps are believed to arise from mucosal hyperplasia and inflammation. A retrospective study conducted by Wang et al. [5] has linked factors such as positive family aggregation, positive serum immunoglobulin E (sIgE), and higher meat intake are associated with an increased risk of colorectal polyps. While these associations do not necessarily imply that diet or bowel habits directly cause polyps in children, they suggest that these factors may play an indirect role in the pathogenesis of the condition.

Even though juvenile rectal polyps are the most common cause of rectal bleeding in children, other conditions, such as anal fissures, inflammatory bowel disease (IBD), and infectious colitis, can also present with rectal bleeding. Furthermore, when evaluating rectal polyps, it is crucial to distinguish between a solitary juvenile polyp and a polyposis syndrome, as the latter may involve multiple lesions with an increased risk of complications, including malignant transformation if left untreated [2].

Diagnosis is typically established through colonoscopy, with definitive confirmation by histopathological examination. While endoscopic polypectomy remains the first-line treatment for juvenile rectal polyps, surgical excision is indicated when the polyps are large, when there is significant bleeding or prolapse, or if technical difficulties preclude complete endoscopic removal. Additionally, surgical excision may be preferred in cases where there is suspicion of complications or malignant potential.

Here, we present a case of juvenile polyps in a 13-year-old boy, highlighting the clinical presentation, diagnostic process, and management.

## Case presentation

A 13-year-old boy presented to the National Institute of Ayurveda Hospital in Jaipur with complaints of chronic bowel evacuation difficulty and episodes of intermittent painless rectal bleeding. He reported that these symptoms had persisted for five years and had become more frequent over the past year. Additionally, he described occasional prolapse of a rectal mass over the past four years, often accompanied by abdominal pain.

The patient initially noticed difficulty in defecation, which was temporarily relieved with laxatives. Subsequently, he began to observe episodes of blood loss in the toilet following defecation. Various general practitioners diagnosed his condition as piles and provided symptomatic treatment. However, these symptoms recurred over time. Over the last year, the prolapse of a rectal mass after defecation became more prominent, accompanied by increasing abdominal pain. Despite multiple consultations by the general practitioners, the patient continued to be misdiagnosed with piles and was managed symptomatically.

The condition worsened, prompting the patient's parents to conduct a personal examination of the mass following defecation. They identified a substantial, lobulated mass protruding from the anus. Although the mass was manually reducible, the patient continued to experience abdominal pain, particularly during its prolapse. This necessitated further evaluation, leading the patient to seek consultation at the Anorectal Outpatient Department of the National Institute of Ayurveda, Jaipur.

On digital rectal examination, multiple large pedunculated masses were felt on the posterior rectal wall, apparently originating above the anorectal junction. The anal sphincter tone was normal. Visual examination by proctoscopy revealed polypoid growths originating above the anorectal junction, without active bleeding. This warranted further evaluation by colonoscopy.

On examination, vital signs were stable, and abdominal examination revealed tenderness in the lower abdomen. Laboratory investigations were within normal limits with hemoglobin level at 11.2 g/dL (normal range: 13-17 g/dL) and hematocrit 34.4% (normal range: 40%-50%) (Table 1).

**Table 1:** Laboratory investigations. μL, microliter; dL, deciliter; fL, femtoliter; pg, picogram; mm, millimeter; RDW-CV, red cell distribution width-coefficient of variation; RDW-SD, red cell distribution width-standard deviation; WBC, white blood cell

Test	Result	Reference interval
Total red blood cell	4.57 x 10^6/μL	4.5-5.5 x 10^6/μL
Hemoglobin	11.2 g/dL	13-17 g/dL
Hematocrit	34.40%	40%-50%
Mean cell volume	74.8 fL	83-101 fL
Mean corpuscular hemoglobin	24.5 pg	27-32 pg
Mean corpuscular hemoglobin concentration	32.7 g/dL	31.5-34.5 g/dL
RDW-CV	12.70%	11.6%-14.0%
RDW-SD	34.70%	39%-46%
Total WBC	6.23 x 10^3/μL	4.0-10.0 x 10^3/μL
Differential leucocyte count		
Neutrophils	50.20%	40%-80%
Lymphocytes	40.30%	20%-40%
Eosinophils	2.10%	0%-6%
Monocytes	6.90%	2%-10%
Basophils	0.50%	0%-2%
Erythrocyte sedimentation rate	5 mm/hour	<10 mm/hour
Clotting time	6.20 minutes	5-10 minutes
Bleeding time	3.36 minutes	2-7 minutes
Random blood glucose	95 mg/dL	90-140 mg/dL

**Figure 1:**
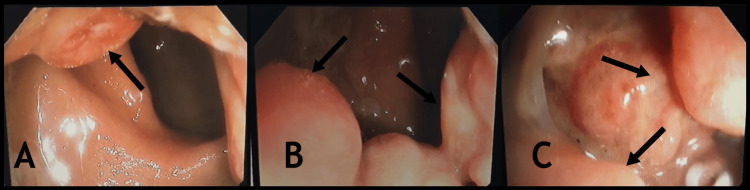
Colonoscopy images showing multiple polyps of varying sizes in the lower rectum. (A) Polyp at the 11 o’clock position. (B, C) Large lobulated polyp on the posterior wall of the lower rectum.

A colonoscopy performed the day after the initial consultation revealed multiple polyps of varying sizes in the distal rectoanal region. The surrounding mucosa appeared inflamed and edematous, raising suspicion for pseudopolyps (Figure 1). Since the polyps were significantly large and were causing significant symptoms, they were scheduled for surgical excision and histopathological examination.

Surgical intervention

**Figure 2:**
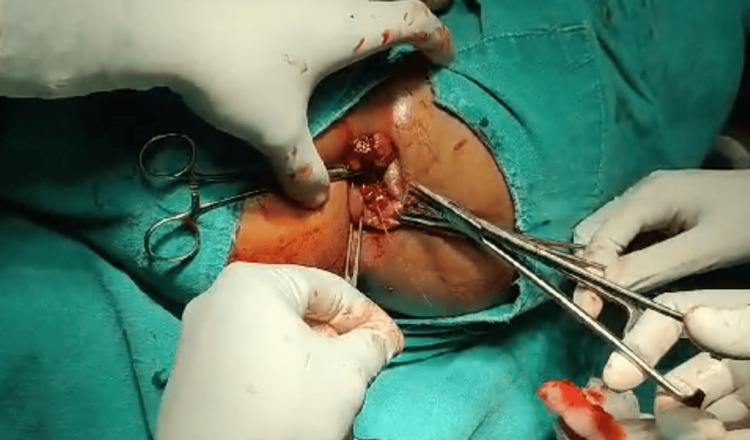
Intraoperative image showing the excision of rectal polyps.

**Figure 3:**
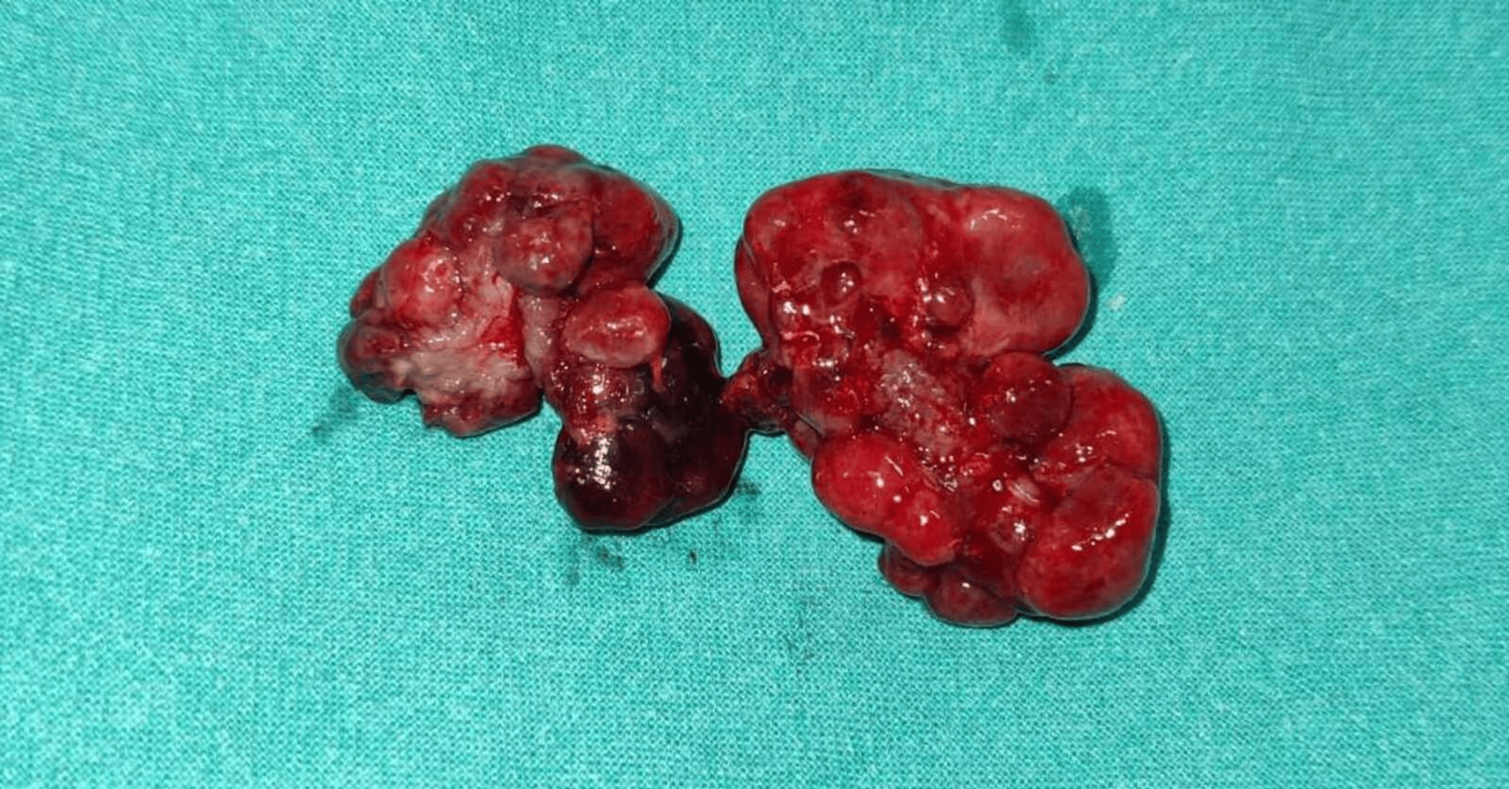
Excised polypoid masses.

Surgical excision of the mass was performed under spinal anesthesia, with standard cardiopulmonary monitoring. Two large polyps located at the 7 o'clock and 11 o'clock positions were excised sequentially. The procedure involved initially transfixing the base and ligating it with 2-0 Vicryl, followed by excision of the mass (Figures 2, 3). Subsequently, the smaller polyps located at the 5 o'clock and 9 o'clock positions were excised. Hemostasis was achieved, and the rectal mucosa was examined for any remaining polyps. The timeline of the intervention and postoperative care is detailed in Table 2.

**Table 2:** Chronological timeline of diagnosis and surgical intervention.

Date	Clinical findings	Therapeutic Intervention
05/02/24	Visited the hospital with complaints of difficulty in bowel evacuation, intermittent bleeding per anum, and a prolapsing mass per anum during defecation, accompanied by abdominal pain.	Routine blood investigations were done and was planned for colonoscopy.
06/02/24	Complaints as previous	Colonoscopy revealed multiple polyps in the lower anorectal region.
15/02/24	Complaints persisting	The patient was admitted and planned for excision under spinal anesthesia. Antibiotic prophylaxis was given with 1 g Ceftriaxone and gentamicin 80 mg intravenously.
16/02/24	Mild pain present	Excision was done mass was sent for biopsy. Parenteral administration of anti-inflammatory drugs was given, and IV antibiotics were continued for two days.
17/02/24	Mild tolerable pain in the abdomen	The same medication regimen was continued.
19/02/24	Bowel movement resumed; minimal postoperative pain	Anti-inflammatory drugs and prophylactic antibiotics were continued.
20/02/24	The patient stable with minimal pain and slight bleeding per anum	The patient was discharged.

Histopathological findings

**Figure 4:**
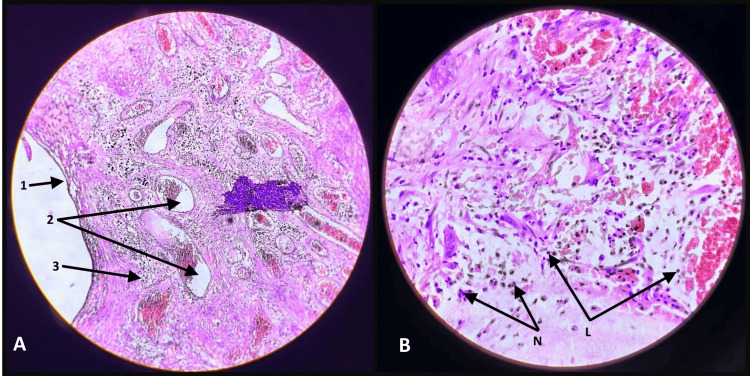
Histological microphotograph of the rectal polyp with cystically dilated glands filled with mucus and lamina propria with inflammatory infiltrates. (A) (1) Simple columnar epithelium consistent with rectal mucosa lining the surface of the polyp; (2) numerous cystically dilated, mucus-filled glands lined by cuboidal epithelium; (3) an inflamed lamina propria infiltrated with inflammatory cells. (B) Interglandular stroma displaying variably sized mucin pools and mixed inflammatory infiltrate including lymphocytes (L) and neutrophils (N).

The biopsy of the excised mass revealed a polyp with focal ulceration of the overlying surface epithelium. Various-sized glands lined by cuboidal epithelium were seen, some of which were cystically dilated and filled with mucus. These glands were separated by inflamed and edematous stroma (Figure 3). The interglandular spaces contained variable-sized mucinous pools and inflammatory infiltrate, along with many dilated and congested blood vessels. The histomorphology was consistent with a juvenile rectal (retention) polyp with features of torsion and extravasated mucin (Figure 4).

## Discussion

Juvenile rectal polyps are commonly seen in children, typically presenting with painless rectal bleeding. Histologically, they are characterized by the cystic dilation of glandular structures within the inflamed lamina propria [1,3]. In the present case, there was a prolapse of a significant mass per anum, accompanied by abdominal pain, and the pronounced symptoms in this patient necessitated further evaluation. Colonoscopy identified multiple polyps located at the lower rectoanal junction, particularly on the posterior and left lateral walls.

The presence of multiple polyps raised suspicion of a hamartomatous polyposis syndrome. Juvenile polyposis syndrome (JPS) is diagnosed if any one of the following criteria is met: (1) five or more juvenile polyps in the rectum or colon; (2) juvenile polyps found elsewhere in the gastrointestinal tract; or (3) juvenile polyps with a positive family history [6]. Although our patient had no family history, literature suggests that approximately 25% of JPS cases arise without a genetic predisposition [2]. Moreover, some studies, including Giardiello et al., propose that the presence of three or more juvenile polyps in the colon or rectum may warrant a diagnosis of JPS [7]. Histologically, JPS is characterized by non-dysplastic normal epithelium, accompanied by an abundance of elongated, cystically dilated, mucus-filled glands within the lamina propria. This lamina propria is infiltrated by myofibroblasts, fibroblasts, and macrophages. Histologically, JPS is characterized by non-dysplastic epithelium, with abundant elongated, cystically dilated, mucus-filled glands within the lamina propria. The stroma often shows the presence of myofibroblasts and fibroblasts, as well as infiltration by macrophages. However, a diagnosis of JPS in this case remains uncertain, as the polyps were confined to the lower rectum, and the patient did not exhibit systemic manifestations often associated with extensive disease, such as hypoproteinemia, malnutrition, or electrolyte imbalance [6]. In this case, several common clinical manifestations of JPS were observed, including rectal bleeding, anemia, abdominal pain, and prolapse of mass per anum [8,9]. Histologically, these resemble solitary juvenile rectal polyps, with glandular densities being slightly more pronounced in JPS [10]. Another possibility that was explored was the scenario of pseudopolyps. Pseudopolyps are benign, non-neoplastic lesions that arise from the mucosa due to repeated episodes of inflammation and mucosal regeneration, commonly observed in IBDs such as ulcerative colitis or Crohn's disease [11]. They may be islands of surviving mucosa situated between ulcerated areas, giving them a polyp-like appearance or as loose tags of mucosal tissue between ulcerated regions [11]. They are classified into *inflammatory polyps*, which develop during the inflammatory process characterized by the formation of granulation tissue in certain intense foci, and *post-inflammatory polyps*, which arise during the healing phase through re-epithelialization and excessive regeneration [11].

Inflammatory polyps are composed of granulation tissue lacking an epithelial lining and infiltrated by inflammatory cells. Post-inflammatory pseudopolyps contain normal or hyperplastic glandular epithelium, muscularis mucosae, and fibrovascular submucosa with minimal inflammation [11,12]. Clinical symptoms like bleeding per rectum and abdominal pain are common to both IBD and juvenile polyps, necessitating histological confirmation for accurate diagnosis. In this case, histopathological examination confirmed a juvenile rectal polyp without dysplasia or malignancy. The polyp showed cystic dilation of glandular structures consistent with juvenile pathology. Uncommonly, torsion of the polyp and extravasation of mucin were noted, complicating the presentation and emphasizing the critical role of histopathology in guiding diagnosis and management.

A critical aspect of this case is the delay in accurate diagnosis. Despite the patient's clinical presentation and age being strongly indicative of a juvenile rectal polyp - a common cause of pediatric rectal bleeding - the initial evaluation by general practitioners led to a misdiagnosis of hemorrhoids. This misdiagnosis, coupled with the absence of an early, targeted digital rectal examination, significantly postponed the correct diagnosis. Had a thorough digital rectal exam been performed during the initial evaluations, the polyp would likely have been identified sooner. Such a delay not only allowed the polyp to grow larger but also increased the risk of repeated trauma or irritation over time. Because the rectal bleeding was intermittent, the general practitioners may have underestimated its clinical significance, opting not to refer the patient to a pediatric gastroenterologist. This case, therefore, highlights the need for increased clinical vigilance and a proactive approach, including early endoscopic referral and digital rectal examinations in pediatric patients with persistent rectal bleeding.

Additionally, the child’s predominantly low-fiber, high-fat Western diet may have contributed to constipation and increased straining factors that might exacerbate mucosal stress and promote the development of larger polyps. Although the exact mechanism remains unclear, such dietary patterns are recognized as potential contributors to an unfavorable colonic environment.

Endoscopic polypectomy is the first-line intervention for symptomatic juvenile polyps [1,2]. However, surgical excision is warranted for larger polyps, especially those associated with significant bleeding or prolapse, as seen in this case. Surgical removal not only alleviated the patient’s symptoms but also enabled definitive histopathological diagnosis. Postoperatively, the child has remained asymptomatic.

Given the presence of multiple polyps and the associated risk of malignancy [13], particularly in cases of JPS, the parents were advised to bring the child for annual surveillance and follow-up.

## Conclusions

This case highlights the importance of considering juvenile polyps in the differential diagnosis of rectal bleeding in pediatric patients, and the potential complications that can arise, such as torsion and mucin extravasation. The delayed diagnosis in this case underscores the crucial need for a careful clinical assessment, particularly when symptoms persist or worsen despite standard treatments aimed at more common conditions like hemorrhoids. Early evaluation, especially by a pediatric gastroenterologist, is essential for ensuring accurate diagnosis and timely intervention. Such a specialized assessment not only facilitates effective symptom management but also helps prevent complications. Histopathological examination remains essential in confirming the diagnosis and guiding treatment.
